# Comparison of plasma substance P concentrations in the blood of healthy male and female German Simmental calves

**DOI:** 10.1186/s12917-024-04010-1

**Published:** 2024-05-24

**Authors:** Anna Landinger, Yury Zablotski, Gabriela Knubben-Schweizer, Theresa Tschoner

**Affiliations:** Clinic for Ruminants with Ambulatory and Herd Health Services, Centre for Clinical Veterinary Medicine, LMU Munich, Sonnenstrasse 16, 85764 Oberschleissheim, Germany

**Keywords:** Dairy calves, Fleckvieh, Pain, Pain Parameter, Reference range, Sex

## Abstract

**Background:**

The recognition of pain is a major problem in cattle, as they are stoic animals which strongly mask their pain. Among objective parameters to assess pain in cattle is substance P (SP), a neurotransmitter which is involved in the pain pathways. Research about SP concentration in calves focus on painful procedures, such as castration and dehorning. Basic research work is lacking; evaluation of SP concentrations in healthy calves and possible differences between sexes have not been published yet. The objectives of this study were to (1) describe SP concentrations in healthy male and female calves of the German Simmental breed to establish benchmarks of orientation, (2) compare SP concentrations between male and female calves, and (3) assess differences in SP concentrations between calves and adult cows. A total of 44 male and 49 female calves aged 14 to 21 days (17.1 ± 2.2 days) were included in this study. Blood samples were taken at 06:00 a.m. from the jugular vein, followed by a clinical examination. SP concentrations were analyzed using a commercial ELISA kit. Differences in SP concentrations according to laboratory parameters, and correlation of SP concentrations with different parameters were assessed.

**Results:**

Median SP concentrations in the blood plasma were 516 pg/ml (Interquartile Range 320 pg/ml, range 229–1615 pg/ml) in calves. Median SP concentrations differed significantly between male and female calves (554 pg/ml for male, and 489 pg/ml for female calves, respectively). There was no significant difference in animals with laboratory findings within reference ranges and those with mild deviations from reference ranges. There was a positive correlation between SP concentrations and leucocyte count, which was significant. SP concentrations were significantly lower in calves compared with a dataset of adult cows, which has been published previously.

**Conclusion:**

Due to the high interindividual differences in SP concentrations, it is hard to establish benchmarks for orientation. Sex has a significant influence on SP concentrations. Research work should preferably be done in animals of the same sex. Also, animals should be within the same age range (adults or calves), as age seems to have an influence on SP concentrations.

**Supplementary Information:**

The online version contains supplementary material available at 10.1186/s12917-024-04010-1.

## Background

In calves, zootechnical procedures such as castration [1] and dehorning [2] are routinely performed. Attitudes towards pain in animals are changing [3] and the interest to reduce pain in context with castration and dehorning [1] as well as general public concern with cattle welfare is increasing [3, 4]. Pain assessment in animals can be done either by measurement of general body functions, physiological responses, or behavior [3]. Assessment of behavior in cattle in terms of pain recognition is more difficult than in other animals, as cattle are stoic animals which hide their pain in front of predators [5, 6]. The measurement of physiological responses to stress or pain (for example cortisol) or other markers (such as acute phase proteins) is termed objective pain assessment [6], and is superior to subjective pain assessment as it is not dependent on the observer’s experience and opinion [6, 7]. In 2008, substance P (SP) was described as an objective and sensitive biomarker for pain in cattle undergoing castration. Contrary to cortisol concentrations, which increased in the blood plasma of castrated as well as sham-castrated calves, SP concentrations only increased in castrated calves [8]. SP is a neurotransmitter composed of 11 amino acids [9–12]. SP has a role in processing noxious sensory information to the brain [13] and is released from the neurons of the spinal ganglion as a reaction to either a thermal, mechanical, or chemical stimulus [14]. Whereas basic research about SP is published in human medicine, it is missing in veterinary medicine. A study evaluating SP concentration in healthy adult German Simmental cows has only been published recently [15]. Nevertheless, studies about SP concentrations in calves undergoing different painful procedures such as castration [8, 16], dehorning [17, 18], and umbilical surgery [19] have been published and SP has been described and used as an objective biomarker for pain. In these studies, small sample numbers have been used [8, 17–19] and high variations of SP concentrations between individual animals could be shown [8, 19]. In all recent studies, SP concentrations were determined in calves which were either submitted to a stress- and/or a painful stimulus [8] and/or which were treated with non-steroidal anti-inflammatory drugs [18, 19]. Also, calves were not sorted by sex [18, 19], even if studies done with laboratory animals indicate that testosterone levels influence SP concentrations in male animals [20, 21]. To the knowledge of the authors, there are no studies to date describing the plasma substance P concentrations (PSPC) in the blood plasma of healthy male and female calves which were kept in their physiologic surroundings and not exposed to any treatment, stress, or pain. The objectives of this study were (1) to describe SP concentrations in the blood plasma of healthy calves of the German Simmental breed, (2) to compare the SP concentrations between male and female calves, and (3) to compare the SP concentrations between calves and an existing dataset of adult cows [15] regarding age.

## Results

### Animals

Age, birth weight, and colostrum intake in calves is presented in Table 1. Findings in individual animals is given in Appendix 1.

**Table 1:** General findings, findings of clinical examination according to Dirksen et al. (1979), and laboratory findings of selected blood parameters in 44 male and 49 female calves of the German Simmental breed sampled for the assessment of substance P concentrations. Parameters are presented as mean and standard deviation (SD). Ranges are given in brackets

	Total (*n* = 93)	Male Calves (*n* = 44)	Female Calves (*n* = 49)
**Parameter**			
**General Findings**			
**Age (d)**	17.1 ± 2.2 (14–21)	17.1 ± 2.1 (14–21)	17.1 ± 2.3 (14–21)
**Birth Weight (kg)** ^1^	42.8 ± 5.8 (28 ± 58)	44.6 ± 4.9 (32–58)	41.2 ± 6.2 (28–54)
**Colostrum Intake (L)** ^2^	2.5 ± 0.9 (0.5–7.0)	2.7 ± 1.1 (0.5–7.0)	2.3 ± 0.7 (0.5–4.0)
**Findings of Clinical Examination**			
**Temperature (°C)**	39.0 ± 0.3 (38.1–39.5)	39.0 ± 0.3 (38.1–39.4)	39.0 ± 0.3 (38.1–39.5)
**Heart Rate (beats/minute)**	137.1 ± 19.4 (100.0 ± 176.0)	134.9 ± 17.7 (108–168)	139.0–20.8 (100–176)
**Respiratory Rate (breaths/minute)**	38.2 ± 6.2 (20.0–49)	38.8 ± 6.8 (20–49)	37.6 ± 5.7 (24–48)
**Findings of Laboratory Analysis**			
**Leucocytes** (4–10 × 10³/µl)	9.8 ± 2.8 (3.2–14.9)	10.0 ± 2.7 (5.3–14.9)	9.6 ± 2.9 (3.2–14.7)
**PCV** ^**3**^ (30–36%)	35.3 ± 2.8 (27.9 ± 40.0)	35.5 ± 2.6 (28.5–40.0)	35.2–2.9 (27.9–39.7)
**Hemoglobin** (10–13 g/dL)	11.4 ± 0.9 (9.0–13.1)	11.5 ± 0.9 (9.6–13.1)	11.4 ± 1.0 (9.0–12.9)
**Total Protein** (40–80 g/L)	57.2 ± 5.1 (47.5–71.8)	57.3 ± 5.4 (47.5–71.8)	57.1 ± 4.8 (47.6–69.4)
**GSPHX** ^**4**^ (> 250 g/Hb)	572.5 ± 144.7 (297.0–995.1)	582.7 ± 140.6 (330.0–995.1)	563.3 ± 149.2 (297.0–983.7)

^1^Birth weight is missing in *n* = 1 female calf, ^2^Colostrum Intake is missing in *n* = 6 male and *n* = 3 female calves ^3^Packed Cell Volume ^4^Gluthathione Peroxidase

### Findings of clinical examination

Findings of the clinical examination of the calves are given in Table 1. Faeces was slightly loose in 12 calves (12.9%, *n* = 3 for male and *n* = 9 for female calves), physiologic in 76 calves (81.7%, *n* = 39 for male and *n* = 37 for female calves), and slightly solid in 5 calves (5.4%, *n* = 2 for male and *n* = 3 for female calves). An uncomplicated umbilical hernia was found in 9 calves (9.7%, *n* = 2 for male and *n* = 7 for female calves). The clinical findings in individual animals are presented in Appendix 1.

### Laboratory findings

Laboratory findings in the calves are given in Table 1 and Appendix 2.

### Substance P concentration in male and female calves

**Fig. 1:**
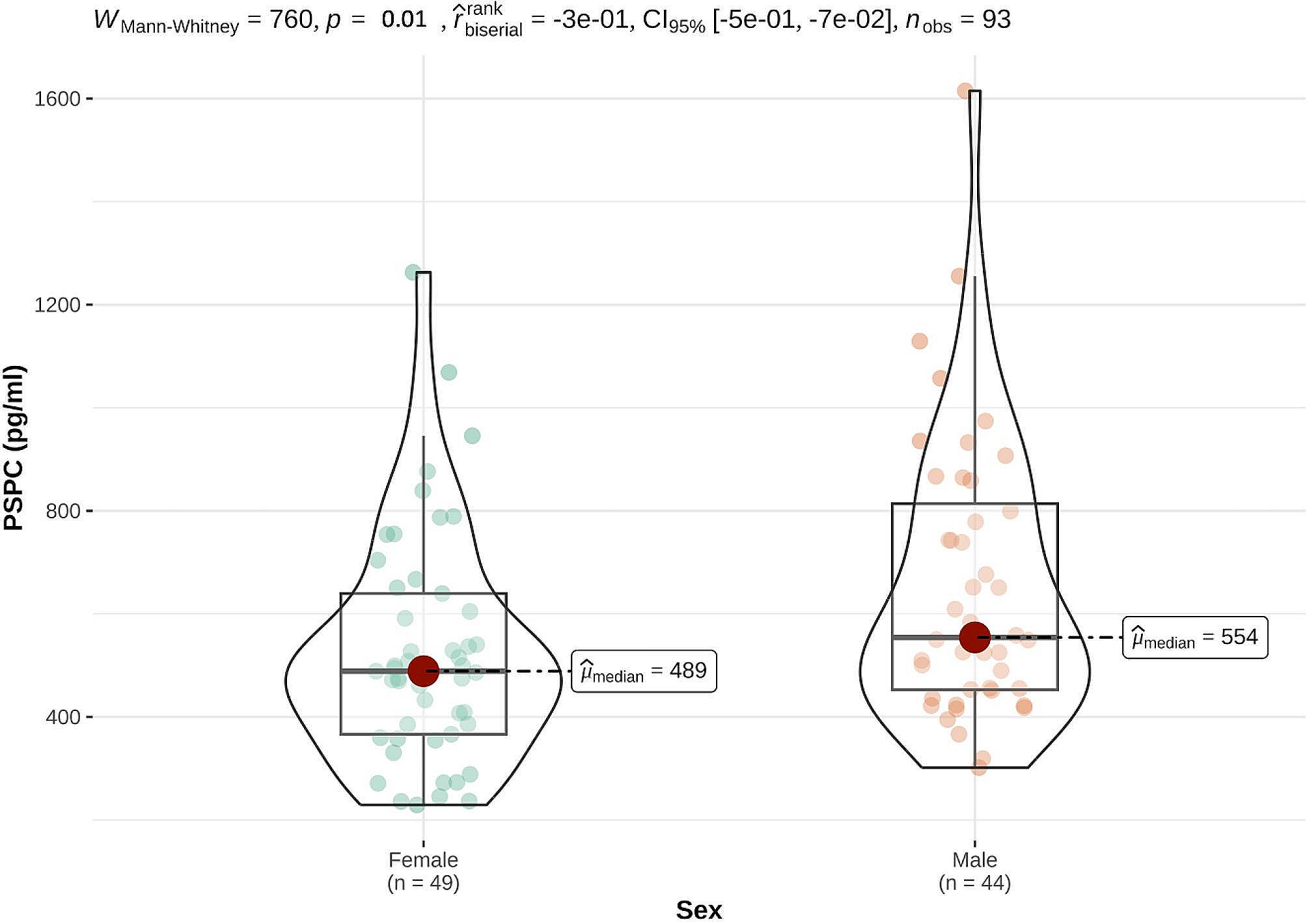
Median plasma substance P concentrations (PSPC) in 44 male and 49 female calves. PSPC were significantly (*p* = 0.01) lower in female compared with male calves

Median and IQR of PSPC in male and female calves, and in total, are presented in Table 2. PSPC in individual animals is given in Appendix 2. PSPC were significantly (*p* = 0.01) lower in female compared to male calves (Fig. 1). PSPC did not differ significantly between animals kept in individual igloos compared with group housing (*p* = 0.6, Table 3).

**Table 2:** Median and Interquartile Ranges (IQR) plasma substance P concentrations (PSPC) in 44 male and 49 female German Simmental calves. All calves were clinically healthy and were exposed to the same surroundings, feeding, and handling. PSPC were significantly (*p* = 0.01) lower in female compared with male calves

PSPC (pg/ml)	Total Calves (*n* = 93)	Male Calves (*n* = 44)	Female Calves (*n* = 59)
**Median**	516	554	489
**IQR**	320	361	273
**Range**	229–1615	302–1615	229–1263

**Table 3:** Comparison of plasma substance P concentrations (PSPC) in 44 male and 49 female German Simmental calves housed either in individual (*n* = 18) or group (*n* = 75) igloos. PSPC are presented as median and Interquartile Ranges (IQR). Group size was 6 ± 2 (2–9 calves). All calves were exposed to the same surroundings, feeding, management, and handling. PSPC did not differ significantly between housing systems

PSPC	Individual Housing			Group Housing		
	Total (*n* = 18)	Male (*n* = 5)	Female (*n* = 13)	Total (*n* = 75)	Male (*n* = 39)	Female (*n* = 36)
**Median**	516	525	499	516	558	488
**IQR**	145	218	104	350	379	317
**Range**	237–859	422–859	237–755	229–1,615	302–1,615	229–1,263

### Comparison of substance P concentrations according to laboratory findings

**Fig. 2:**
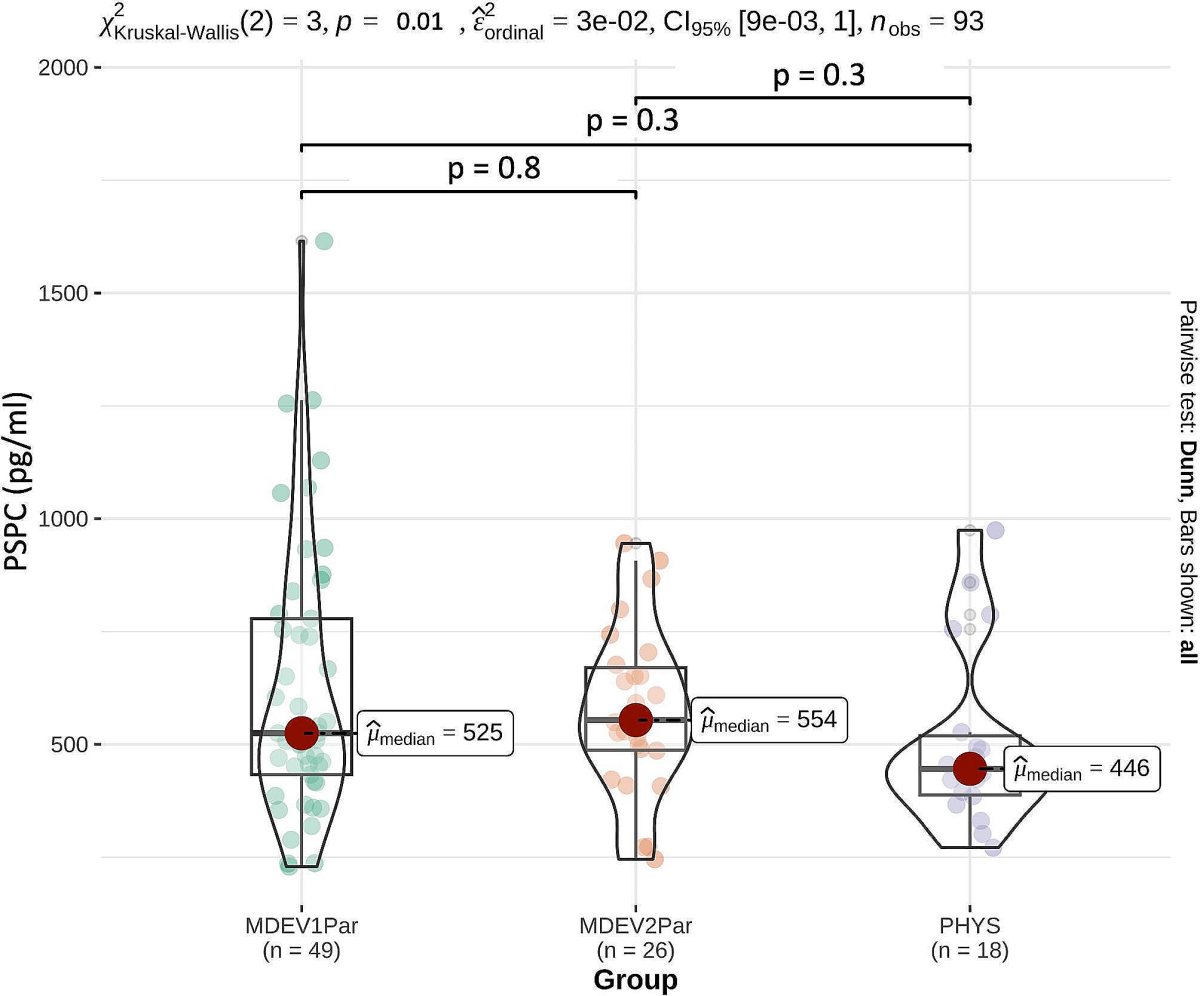
Median plasma substance P concentrations (PSPC) in 18 clinically healthy calves with laboratory findings with no deviations from reference ranges as defined by the Clinic for Ruminants with Ambulatory and Herd Health Services (PHYS), 49 clinically healthy calves with mild deviations in one (MD1Par), and 26 clinically healthy calves with mild deviations in two (MD2Par) laboratory parameters. There were no significant differences in PSPC between groups

PSPC in PHYS, MDEV, MD1Par, and MD2Par are given in Table 4. There were no significant differences between groups (Fig. 2), but a trend (*p* = 0.08) between PHYS and MDEV.

**Table 4:** Comparison of plasma substance P concentrations (PSPC) in 44 male and 49 female German Simmental calves according to laboratory findings of selected blood parameters. PSPC are presented as median and Interquartile Ranges (IQR). Calves were divided into groups PHYS (*n* = 18, clinically healthy with blood parameters within the references determined by the Clinic for Ruminants with Ambulatory and Herd Health services) and MDEV (*n* = 75, clinically healthy with blood parameters showing mild deviations from the reference ranges). MDEV were further divided into animals with either one (MD1Par) or two (MD2Par) parameters deviating from the reference ranges

	Group according to Laboratory Findings			
PSPC (pg/ml)	PHYS (*n* = 18)	MDEV (n ) 75)	MD1PAR (*n* = 51)	MD2PAR (*n* = 24)
**Median**	466	529	525	539
**IQR**	131	301	346	183
**Range**	271–974	229–1,615	229–1,615	246–946

### Plasma substance P concentration in correlation to different parameters

**Fig. 3:**
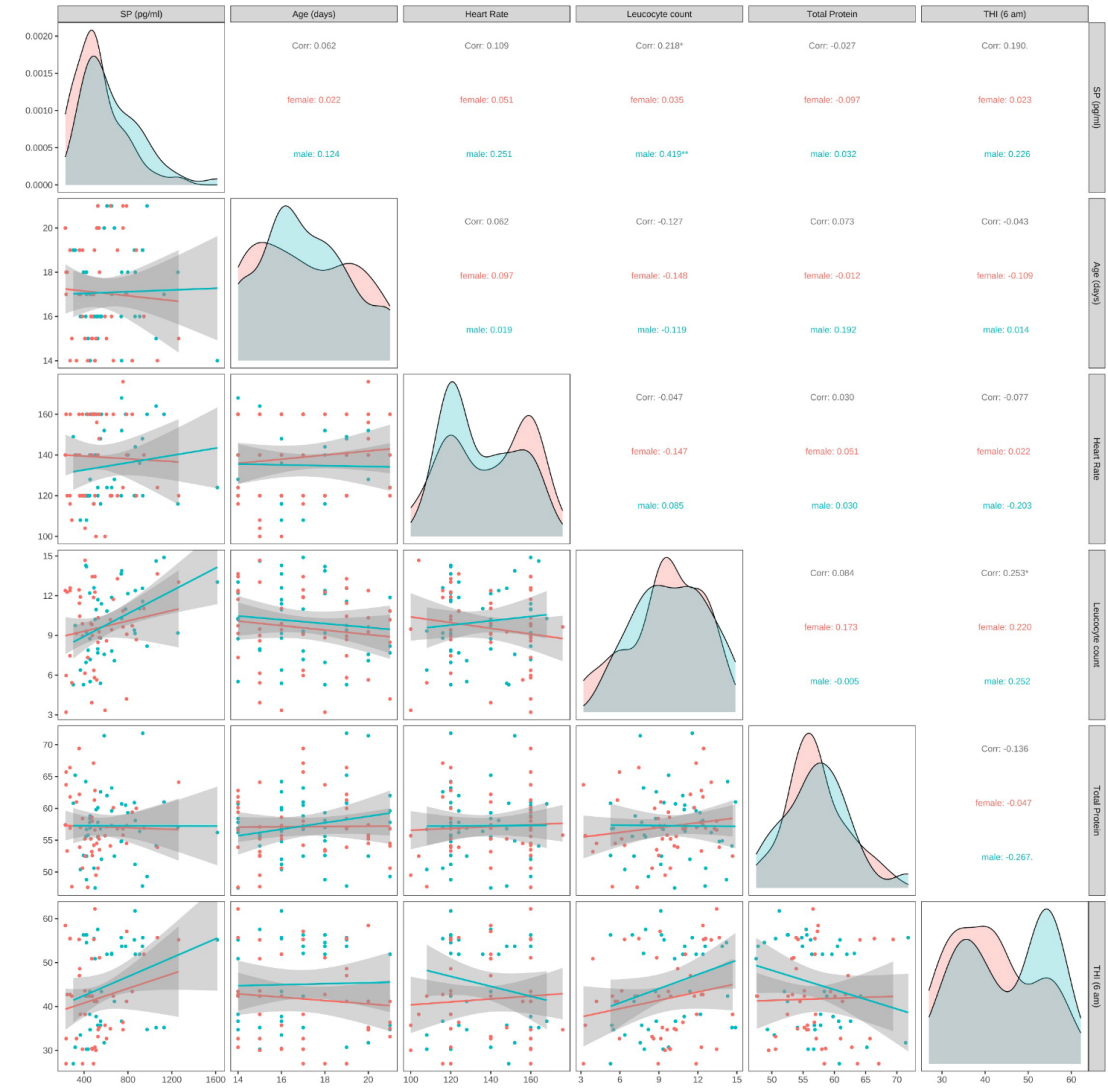
Spearman correlation between plasma substance P concentrations in 93 calves (in black) of the German Simmental breed (*n* = 44 for male (in blue) and *n* = 49 for female (in red) calves) and age in days, heart rate, leucocyte count, total protein, and temperature humidity index (THI). There was a significantly positive correlation between PSPC and leucocyte count, both for total number of calves (rho = 0.218, *p* = 0.05) and male calves (rho = 0.419, *p* = 0.01). Significant codes: 0 *** 0.001 ** 0.01 * 0.05 ‘.’ 0.1 ‘ ’ 1. Spearman rank correlation can be interpreted by Funder’s rules [50]): *r* < 0.05 – Tiny, **0.05 < = ***r*** < 0.1** - Very small, **0.1 < = ***r*** < 0.2** – Small, **0.2 < = ***r*** < 0.3** – Medium, **0.3 < = ***r*** < 0.4** – Large, **r > = 0.4** - Very large

There was no correlation between PSPC and age in days (rho = 0.062), PSPC and heart rate (rho = 0.109), PSPC and total protein (rho = -0.027), and PSPC and THI (rho = 0.190). There was a significantly positive correlation between PSPC and leucocyte count, both for total number of calves (rho = 0.218, *p* ≤ 0.05) and male calves (rho = 0.419, *p* ≤ 0.01). Correlation between PSPC and different parameters for total number of calves, as well as for female and male calves, is presented in Fig. 3.

### Comparison of substance P concentrations in calves and adult cows

PSPC in calves were compared with a dataset of PSPC assessed from the jugular vein in 52 adult cows (mean and SD 1,087 pg/ml ± 436 pg/ml, median 984 pg/ml [15]. Median PSPC of adult cows were significantly higher than PSPC of total calves (*p* < 0.01), and female calves (*p* < 0.01) and male calves (*p* < 0.01), respectively.

## Discussion

The present study was done to evaluate the PSPC in healthy male and female German Simmental cattle, and to compare PSPC between sexes. The study population is representative of calves in Bavaria, Germany.

### Sampling method

As described for [15], blood samples for the assessment of a pain marker were taken invasively from a vein, which in itself is a painful procedure resulting in nociception and thus an increase of PSPC. Therefore, this could be seen as a limitation for the present study. Non-invasive sampling for SP concentrations via saliva samples has been described [22]. For saliva samples, aprotonin, a protease inhibitor which keeps SP from degrading [23], can only be added to samples after centrifugation, due to sampling techniques [22]. Harvesting of plasma after centrifugation should be done within 1 [23] or 2 [19, 24] hours after sampling, with samples being spiked with aprotonin and kept on ice until centrifugation [19, 24]. As samples for the present study were transported to the Clinic for Ruminants with Ambulatory and Herd Health Services for centrifugation, PSPC in saliva samples would not have been representative anymore. SP concentrations show a delayed onset of excitation of the dorsal horn (20 to 40 s), and a slow response (30 to 90 s) [25–27]. Therefore, blood sampling via punctation, which takes less than 30 s, should not have influenced our results.

### Influence of different parameters on plasma substance P concentrations

PSPC concentrations are described to be influenced not only by pain [8], but also by stress [28–30], and inflammation [11, 31]. To exclude the influence of stress on our results, calves were not taken from their physiologic surroundings for the duration of the trial, and feeding, management, and handling were not changed. Heart rate of calves during clinical examination ranged from 100 to 176. As heart rate significantly increases in cattle due to stress [32], it can be assumed that blood sampling as well as clinical examination resulted in stress for the animals. However, stress should not have influenced our results, as there was no correlation between heart rate of calves and PSPC. Also, blood samples were taken prior to clinical examination right after entering the animal’s igloo to reduce the influence of stress on the PSPC. Blood samples were taken through all seasons, minimizing the influence of seasonal changes on our results. To exclude the influence of heat stress on the present results, THI was assessed for all sampling times. As there was no correlation between THI and PSPC, influence of heat stress on the present results can be excluded. Breed of animal should also be considered as a confounder. A previous study showed significantly smaller immunoreactive areas for SP in the corpus abomasi in German Holstein than in German Simmental cows [33]. However, another study found no significant difference in SP concentrations in the blood serum between these two breeds [34]. As for the present study, only calves of the German Simmental Breed were sampled, we can’t make a statement about the influence of breed on PSPC.

### Clinical examination and laboratory findings

Clinical examination was done following a strict protocol [35] by either AL or TT, to minimize inter-observer bias. Additionally, health status of animals was tracked by the calves’ care takers for two weeks prior and one week following the trial to exclude animals reported to be sick within this period. As the authors did not examine the animals during that time but were dependent on the observation and assessment of the (well-trained and experienced) care takers, this is another possible confounder. Selected blood parameters (PCV, hemoglobin, leucocyte count, total protein, and glutathione peroxidase) were taken to assess the health status of calves. However, in calves which were assessed as healthy via clinical examination, only 19.4% showed no deviations from the reference ranges defined by our clinic for any of the investigated laboratory parameter. This discrepancy between clinical and laboratory findings is in accordance with previously published data in adult cows [15]. Studies show that reference ranges of laboratory parameters always include a 5% false-positive rate (incorrectly diagnosed as being diseased) [36], and that deviations from reference ranges occur in 2 to 24% of clinically healthy cattle [37]. Therefore, to be able to include a higher number of calves into the statistical model, clinically healthy animals with only mild deviations were not excluded from the data; however, this is a limitation of the study. Even if there was no significant difference between PHYS and MDEV, there was a positive correlation between PSPC and leucocyte count, which was significant. SP is described to be an inflammatory mediator [38]. SP concentrations increase during an inflammation, and chemotaxis of neutrophile and eosinophile granulocytes, as well as migration of inflammatory cells to the tissues is controlled by SP [11, 31, 39]. Research about the role of SP during inflammatory processes in cattle is lacking. The results of our study suggest that SP concentrations are influenced by inflammatory cells in cattle, which limits the use of our data as orientation benchmarks for PSPC in a healthy population of calves.

### Plasma substance P concentrations in calves

Median and IQR PSPC was 516 pg/ml in calves. These data are comparable to some studies. Coetzee et al. found mean baseline PSPC of 507.46 ± 92.22 pg/ml and 506.42 ± 76.89 pg/ml in untreated control and trial calves [8]. Mayer described PSPC of 443.49 ± 90.43 pg/ml in calves 2.5 h following tail docking and 366.84 ± 96.41 pg/ml in a control group [22]. Tschoner et al. published median PSPC of 690.0 pg/ml and 560.3 pg/ml in two groups of calves 1.5 following administration of meloxicam [19]. However, in other studies, PSPC are not comparable with the present data. Mean (SE) PSPC were 164.47 (12.30) pg/ml in a control group of calves [40]; another study found mean (SE) PSPC of 247.7 (13.9) pg/ml in meloxicam and 340.5 (23.0) pg/ml in saline treated calves following band castration, and 267.9 (11.2) pg/ml in meloxicam and 314.7 (13.4) pg/ml in saline treated calves following surgical castration [41]. In most studies, PSPC are evaluated either following a painful and/or stressful stimulus, such as castration or dehorning, or following the application of an analgesic. Coetzee et al. showed that PSPC in calves were estimated to be 0.5 times less following the administration of meloxicam compared with a placebo after dehorning [42]. Therefore, comparing our results with a population of untreated, unstressed calves which were not experiencing a painful stimulus is difficult. Also, a high interindividual variation of PSPC has been described in calves [8, 19], as well as in adult cows [15, 24], which is in accordance with the present data and reduces its use as orientation benchmarks.

### Influence of sex on plasma substance P concentrations

PSPC between male and female calves differed significantly, with female calves showing lower PSPC compared with male calves. Previous research work did not indicate a (significant) difference in PSPC between sexes. This could be due to most studies describing PSPC during different methods of castration in male calves [8, 16, 41]. Even in research work about dehorning in calves, populations are either only male [17, 43], or female [40], allowing no comparison between sexes. Stock et al. found no effect of sex on SP in disbudded calves [44]. Tschoner et al. investigated PSPC during umbilical surgery in 17 male and 2 female calves but did not assess differences according to sex [19]. Trials in hamsters showed that testosterone regulates SP levels in those areas of the brain, which also regulates the mating behaviour [20]. In rats, anterior pituitary levels of SP-like immunoreactivity were higher in male compared with female rats; castration on day one of life resulted in a decrease of the levels of SP-like immunoreactivity, which could be restored by replacement of neonatal testosterone [21]. These findings support the theory that testosterone might be responsible for the difference in PSPC in our study population. Further research should investigate the difference in PSPC between adult cows and bulls.

### Influence of age on plasma substance P concentrations

PSPC of calves were compared to a previously published dataset of adult cows. Cows were 3.4 to 9.1 years old. PSPC were significantly higher in adult cows compared with calves. Dockweiler et al. already published that SP concentrations were significantly higher in 6-months-old compared with 8-week-old calves, regardless of method of castration [16]. These findings are not in accordance with findings in other species. In rats, SP concentrations in the brain nuclei of 4 to 5 months old rats were higher compared with 24 to 26 months old rats [45]. In human tears, SP-like immunoreactivity concentration did neither vary by age, nor by gender [46]. Mean concentrations of immunoreactive SP in cerebrospinal fluid of fetuses, premature babies, full term newborns, children, and adults differed significantly between all age groups. The authors concluded that the continuous decline towards maturity supports the idea that SP is involved in the neuro-development [47]. As we only took blood samples for the present study, we can’t make a statement about SP and the age difference in concentrations in different matrices. Also, we did not sample further age groups, such as 6-months-old calves, heifers, or first lactation cows. SP plays a role in the immune system and is involved in inflammatory processes [31, 39]. Therefore, it might be possible that PSPC are higher in adult cattle as these have been presented to a higher number of inflammatory challenges. However, as ruminants seem to have a well-developed immune system from birth on [48], the influence of the immune system on PSPC is unclear.

### Benchmarks of orientation for plasma substance P concentrations

A limitation of the present study is the fact that even with assessing PSPC in clinically healthy male and female calves, we can’t provide reference ranges or benchmarks for orientation for PSPC. This has also been described for PSPC in healthy German Simmental cows [15]. Due to the high interindividual differences in PSPC, which has been described earlier [8, 19], it is difficult to establish a range of PSPC in “healthy” animals. Also, as calves with mild deviations from reference ranges in laboratory findings were included in the present data set, this might have influenced the quality of establishing benchmarks for orientation. This is supported by the positive correlation between PSPC and leucocyte count.

## Conclusions

Due to the high interindividual differences in PSPC, and the many factors possibly influencing PSPC (pain, stress, inflammation), single blood samples to assess PSPC are of limited use. PSPC in calves differed significantly between male and female calves. Researchers should be aware of the difference in sexes and should preferably only use animals of one sex for their studies. As age also seems to have an influence on PSPC, age group of animals used for trials should be within a certain range (e.g. cattle, or adult cow). Therefore, even if the present data are of limited use for practitioners, researcher can benefit from this data set regarding the planning of their study population.

## Materials and methods

All experimental procedures in the present study were approved by the ethics committee of the government of Upper Bavaria (reference number Az. 2532.Vet_03-19-72). Considering that the primary research hypothesis of the study involved comparing males and females as two distinct groups, a two-sample t-test was employed to determine the necessary sample size. Anticipating a substantial difference in substance P (SP) concentrations between the sexes, a large effect size of 0.8 (Cohen’s D) was chosen. A power of 90% and a significance level of 5% were also considered. Based on these parameters, a sample size of 34 animals per group was calculated. Taking into account an anticipated dropout rate of approximately 20%, an additional 7 reserve animals were required for each group. Consequently, the experimental design necessitated a total of *n* = 82 animals, with 41 animals needed for each group. Data collected from December 2019 to December 2021 were included in this study. A total of 73 male and 88 female were sampled for the present study, with 28 male and 39 female calves being excluded due to either clinical symptoms and/or major deviations in the biochemical profiling. One male calf was excluded from the dataset due to its PSPC exceeding a value of 3000 pg/ml.

### Animals, housing and husbandry

**Fig. 4:**
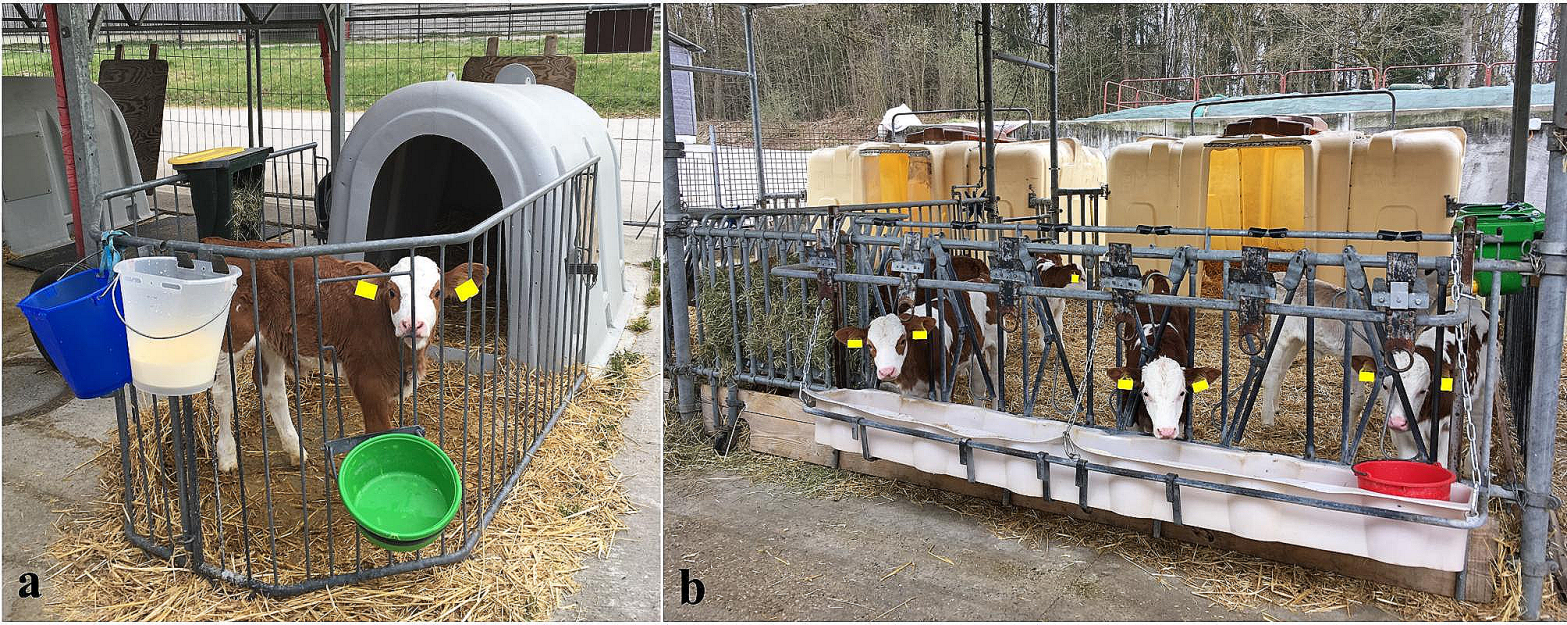
Housing of 93 calves of the German Simmental breed which were sampled to assess plasma substance P concentrations. Calves were either kept in individual igloos (**a**, *n* = 75) or in groups of 2 to 9 (6 ± 2) calves (**b**, *n* = 18). Calves in individual igloos had visual and hearing contact to other calves. Calves had ad libitum access to whole milk, water, hay, and a total mixed ration

A total of 44 male and 49 female calves of the breed German Simmental (≥ 75%, 14 to 21 (17.1 ± 2.2) days old) were included in this study. Calves had to be healthy with no treatment with an antibiotic or non-steroidal anti-inflammatory drug in the last two weeks before and for at least one week after the trial. Colostrum intake as well as birth weight was documented. Information about colostrum intake was missing in 9.7% (*n* = 9) of calves (*n* = 6 of male and *n* = 3 of female calves). Birth weight was missing in 1.1% (*n* = 1) of calves (*n* = 1 for female calves). All animals were kept in their physiologic surroundings for the duration of the study. Calves were either kept in individual igloos (3 m^2^ space per calf, *n* = 75, 80.7%, Fig. 4a) or in small groups (*n* = 18, 19.4%, Fig. 4b). Group size was 6 ± 2 (2–9 calves). Calves either had visual and hearing contact (individual igloos) or visual, hearing, and tactile contact (group housing). Calves which were kept in groups were not rehoused on the day before the trial to reduce stress for the animals before blood sampling. Calves had ad libitum access to whole milk, water, hay, and a total mixed ration. At the day of birth, all calves were treated with 5 ml Alpha-Tocopherolacetet and Natriumselenit (Vitamin E-Selen®, 100 mg/ml + 0.658 mg/ml, cp-pharma), and with Halofuginon (Halocur®, 0,5 mg/ml, MSD, 100 µg per kg of BW) for seven consecutive days. Zootechnical procedures (dehorning or castration) were performed after the study. No animal was euthanized for this study.

### Collection of blood samples for substance P analysis

Blood samples for evaluation of state of health and basic plasma substance P concentration (PSPC) were taken a 06:00 a.m. via punctuation of one Vena jugularis with a 18G needle (BVJ). Blood samples for evaluation of basic PSPC were transferred to an EDTA tube spiked with 9 µl aprotinin. Blood samples were analyzed within two hours at the laboratory of the Clinic for Ruminants with Ambulatory and Herd Health Services. Blood samples for the determination of PSPC were centrifuged (4 °C, 1600 x g for 15 min) and blood plasma was then kept at -20 °C until analysis.

### Laboratory parameters for the assessment of health status

Additional blood samples were taken for assessment of concentrations of PCV (plasma), hemoglobin (plasma), leucocyte count (whole blood), total protein (serum), and glutathione peroxidase (whole blood). Plasma samples were analyzed with the Vetscan® HM5 (Abaxis, Union City, USA), and whole blood samples with the RAPIDPoint® 450 (Siemens, Munich, Germany), and the cobas® c 311 Analyzer (Roche, Basel, Switzerland) for serum samples. Laboratory findings were within the reference ranges defined by the Clinic for Ruminants with Ambulatory and Herd Health Services in 19.4% (*n* = 18) of animals. To be able to include more animals into the statistical analysis, clinically healthy calves with mild deviations from laboratory findings were included into the present study if laboratory findings were within a range of 12–15% of the reference ranges as defined by the clinic for ruminants (presented in brackets): 3–15 × 10³/µl (4–15 × 10³/µl) for leucocyte count, 8–15 g/dl (10–13 g/dl) for hemoglobin, and 26–40% (30–36%) for PCV. Total protein and glutathione peroxidase (GSPHX) were within the reference ranges in all animals. Calves were divided into groups PHYS (*n* = 18, no deviations from reference ranges) and MDEV (*n* = 75, mild deviations from reference ranges). MDEV was further divided into MD1Par (*n* = 49, one laboratory parameter deviating from reference ranges) and MD2Par (*n* = 26, two laboratory parameters deviating from reference ranges).

### Clinical examination

After blood sampling (06:10 am), a clinical examination as described by [35] was performed by one of two authors (AL was trained by TT) in each animal to ensure that the calves were clinically healthy. If an animal was not clinically healthy, it was dismissed from the study.

### Temperature humidity index

To control for heat stress as a possible confounder on PSPC, temperature humidity index (THI) was assessed for each sampling time. Temperature (°C), relative humidity, and THI were recorded every 10 min using a climate sensor (smaXtec animal care, Graz, Austria) which was positioned at the Research- and Teaching Center, using a formula as described by [49]. Data recorded at 06:00 a.m. was included in this study. Due to technical reasons, data for 6.5% of animals (*n* = 6, *n* = 3 each for male and female calves) is missing.

### Age

The dataset of calves of the present study was compared to a previously published dataset of adult cows [15] to assess influence of age on PSPC. Calves were aged 17.1 ± 2 (14 to 21) days. Cows were aged 5.0 ± 1.31 years (3.4–9.1 years), 117 to 239 days in milk (175.0 ± 34.1 days), and number of lactations was 3.2 ± 1.3 (2–7 lactations), with an average daily milk yield of 37.5 ± 4.9 kg (29.5–51.6 kg) [15].

### Substance P analysis

Determination of PSPC was performed as described by [15] with a Substance P ELISA kit (ENZO®, Enzo Life Sciences GmbH, DE). Optical densities were assayed in duplicate, and means were generated for the calculation of concentrations. Lower and upper limits for the quantification of the SP ELISA kit were 167.78 pg/ml and 2,500 pg/ml. The intra- and interassay coefficient of variation was 20%.

### Statistical analysis

The data analysis was conducted using R 4.2.1 (2022-06-23, R Foundation for Statistical Computing, Vienna, Austria). Statistical significance was determined using a *p*-value threshold of < 0.05. A total of 44 male calves and 49 female calves were included in the study; one male was excluded due to being an outlier: calf number 24 exhibited a value exceeding 3000 pg/ml leading to its removal from the dataset. The normality of the data was assessed using the Shapiro-Wilk normality test. Since the data was found to be non-normally distributed, non-parametric tests were employed.

Specifically, the Mann-Whitney U test was utilized to compare the levels of substance P between male and female calves, adult cattle and calves, as well as single and group housing, and groups according to laboratory findings. To compare the substance P concentrations among adult cows and male or female calves, and PHYS, MDEV1Par, and MDEV2Par conditions, the Kruskal-Wallis test followed by Dunn post-hoc tests with Holm *p*-value correction for multiple comparisons was applied.

Pairwise Spearman correlation analyses were performed to assess the associations between SP, age, heart rate, leucocyte count, total protein, and THI (temperature-humidity index) variables. These analyses were conducted both for the entire sample and separately for males and females.

### Electronic supplementary material

Below is the link to the electronic supplementary material.

Supplementary Material 1

Supplementary Material 2

## Data Availability

All data generated or analysed during this study are included in this published article and its supplementary information files.

## Abbreviations

Dl: Deciliter
EDTA: Ethylenediamine tetraacetic acid
e.g.: Example given
ELISA: Enzyme-Linked Immunosorbent Assay
G: Gauge
G: Gram
GSPHX: Glutathione Peroxidase
gHb: Gram Hemoglobin
IQR: Interquartile Range
MDEV: Mild Deviations from Reference Ranges in Laboratory Findings
MD1Par: Mild Deviations from Reference Ranges in 1 Laboratory Parameters
MD2Par: Mild Deviations from Reference Ranges in 2 Laboratory Parameters
min: Minutes
ml: Milliliter
PHYS: Physiologic Laboratory Findings
PCV: Packed Cell Volume
pg: Picogram
PSPC: Plasma Substance P Concentration
rho: Spearman Correlation Coefficient
SD: Standard Deviation
SE: Standard Error
SP: Substance P
THI: Temperature Humidity Index
U: Unit
µg: Microgram
µl: Microliter
°C: Degree Celcius
