# Supplementary material for: Comparison of plasma substance P concentrations in the blood of healthy male and female German Simmental calves
Source: BMC Vet Res. 2024 May 24;20:226. doi: 10.1186/s12917-024-04010-1 (PMC11127320; doi:10.1186/s12917-024-04010-1)
Supplement: Supplementary file 1 — Supplementary Material 1 [file 12917_2024_4010_MOESM1_ESM.docx]

**Appendix 1:** Parameters and clinical findings in 44 healthy male and 49 healthy female calves of the German Simmental breed which were sampled to assess substance P concentrations in the jugular vein. Calves were either kept individually in igloos with visual and tactile contact to other calves, or in group igloos. Housing is given as number of animals per igloo. Missing parameters are indicated as nA (not applicable).

| **Findings in Male Calves** | | | | | | | | | |
| --- | --- | --- | --- | --- | --- | --- | --- | --- | --- |
| **Number** | **Age (d)^1^** | **BW^2^** | **Colostrum^3^** | **Housing** | **T**^4^ | **HR**^5^ | **RR**^6^ | **Faeces^7^** | **Umbilical hernia** |
| 1 | 14 | 58 | nA | 7 | 39.3 | 120 | 36 | physiologic | - |
| 3 | 19 | 44 | 3.5 | 4 | 39.2 | 149 | 49 | physiologic | - |
| 4 | 19 | 47 | 4 | 8 | 39.2 | 140 | 40 | physiologic | - |
| 5 | 17 | 43 | 3 | 7 | 39.2 | 108 | 44 | physiologic | - |
| 6 | 15 | 52 | 3 | 7 | 39.2 | 120 | 48 | slightly loose | - |
| 7 | 17 | 41 | 2.5 | 7 | 38.1 | 160 | 40 | physiologic | - |
| 8 | 18 | 39 | 3 | 7 | 39.3 | 120 | 36 | physiologic | - |
| 9 | 18 | 37 | 3 | 7 | 39.1 | 120 | 36 | physiologic | - |
| 11 | 16 | 46 | 3 | 5 | 39.1 | 116 | 36 | physiologic | - |
| 13 | 16 | 44 | 1.5 | 9 | 38.7 | 108 | 36 | physiologic | - |
| 14 | 17 | 50 | 2 | 9 | 39.0 | 160 | 40 | physiologic | - |
| 15 | 16 | 49 | 1.5 | 5 | 38.7 | 120 | 36 | physiologic | - |
| 16 | 16 | 36 | nA | 2 | 39.4 | 160 | 44 | physiologic | - |
| 17 | 14 | 45 | 1.5 | 8 | 38.8 | 140 | 40 | physiologic | - |
| 18 | 18 | 47.5 | 2.5 | 1 | 39.3 | 120 | 36 | physiologic | - |
| 23 | 14 | 45 | 3 | 8 | 39.1 | 140 | 40 | physiologic | - |
| 26 | 15 | 41 | 2 | 1 | 39.1 | 140 | 36 | physiologic | - |
| 31 | 19 | 41.2 | nA | 5 | 39.4 | 148 | 48 | physiologic | - |
| 32 | 18 | 53 | 3 | 5 | 38.7 | 152 | 44 | physiologic | - |
| 33 | 17 | 47 | 3 | 5 | 39.2 | 160 | 44 | physiologic | - |

**Continuing Appendix 1:**

| **Findings in Male Calves** | | | | | | | | | |
| --- | --- | --- | --- | --- | --- | --- | --- | --- | --- |
| **Number** | **Age (d)^1^** | **BW^2^** | **Colostrum^3^** | **Housing** | **T**^4^ | **HR**^5^ | **RR**^6^ | **Faeces^7^** | **Umbilical hernia** |
| 34 | 20 | 32 | 2 | 6 | 38.7 | 128 | 40 | physiologic | - |
| 35 | 16 | 37 | nA | 3 | 38.8 | 116 | 44 | physiologic | - |
| 36 | 14 | 42 | 2 | 1 | 38.8 | 168 | 48 | slightly loose | - |
| 37 | 17 | 47 | nA | 3 | 39.0 | 120 | 44 | physiologic | - |
| 38 | 17 | 41 | 2 | 3 | 38.9 | 160 | 48 | slightly solid | - |
| 39 | 15 | 43 | 2 | 3 | 39.1 | 164 | 48 | physiologic | - |
| 41 | 16 | 48 | 2 | 6 | 38.3 | 160 | 44 | physiologic | - |
| 45 | 14 | 44 | 2 | 3 | 38.9 | 128 | 28 | slightly solid | - |
| 47 | 21 | 48 | 0.5 | 6 | 38.9 | 160 | 44 | physiologic | - |
| 51 | 20 | 46 | 3 | 8 | 39.2 | 152 | 40 | physiologic | - |
| 54 | 21 | 42 | 7 | 1 | 38.5 | 124 | 40 | physiologic | - |
| 55 | 16 | 44 | 3 | 3 | 38.4 | 148 | 40 | physiologic | - |
| 56 | 18 | 46 | 2.5 | 6 | 39.2 | 140 | 44 | physiologic | - |
| 57 | 18 | 49 | 3.5 | 8 | 39.3 | 116 | 36 | physiologic | - |
| 58 | 16 | 41 | 2 | 8 | 39.4 | 120 | 40 | physiologic | - |
| 60 | 21 | 42 | 4 | 6 | 38.9 | 124 | 24 | physiologic | - |
| 61 | 21 | 50 | 3.5 | 6 | 38.9 | 124 | 20 | physiologic | - |
| 63 | 16 | 40 | 2 | 3 | 39.3 | 136 | 32 | physiologic | yes |
| 64 | 18 | 50 | 3 | 3 | 39.3 | 144 | 40 | physiologic | - |
| 67 | 16 | 45 | 3.5 | 3 | 39.2 | 120 | 28 | physiologic | - |
| 70 | 19 | 43 | 3 | 6 | 38.8 | 120 | 32 | physiologic | - |
| 71 | 16 | 49 | 0.5 | 8 | 38.9 | 120 | 28 | physiologic | - |
| 72 | 19 | 41 | 2.5 | 1 | 38.8 | 120 | 32 | slightly loose | - |
| 73 | 14 | 45 | 3 | 3 | 39.2 | 124 | 32 | physiologic | yes |

**Continuing Appendix 1:**

| **Findings in Female Calves** | | | | | | | | | |
| --- | --- | --- | --- | --- | --- | --- | --- | --- | --- |
| **Number** | **Age (d)^1^** | **BW^2^** | **Colostrum^3^** | **Housing** | **T**^4^ | **HR**^5^ | **RR**^6^ | **Faeces^7^** | **Umbilical hernia** |
| 1 | 15 | 28 | 2 | 1 | 38.7 | 100 | 24 | physiologic | - |
| 4 | 17 | 45 | 2.5 | 7 | 39.5 | 120 | 44 | physiologic | yes |
| 6 | 18 | 51 | nA | 8 | 39.3 | 120 | 40 | slightly loose | - |
| 9 | 14 | 37 | 2 | 8 | 39.1 | 116 | 44 | slightly loose | - |
| 10 | 19 | 36 | 3 | 4 | 38.8 | 120 | 36 | physiologic | - |
| 11 | 18 | 41 | 2.5 | 4 | 39.4 | 160 | 36 | slightly loose | - |
| 12 | 17 | 42 | 3 | 4 | 39.2 | 116 | 28 | physiologic | - |
| 13 | 14 | nA | 3 | 1 | 39.4 | 120 | 36 | physiologic | - |
| 14 | 17 | 44 | 3 | 7 | 39.1 | 160 | 44 | physiologic | yes |
| 15 | 16 | 43 | 3 | 7 | 39.3 | 160 | 44 | physiologic | yes |
| 16 | 21 | 51 | 2.5 | 4 | 39.0 | 140 | 40 | slightly loose | - |
| 18 | 19 | 42 | 2.5 | 5 | 38.7 | 120 | 32 | physiologic | - |
| 19 | 19 | 34 | 3 | 5 | 39.4 | 160 | 40 | physiologic | - |
| 21 | 19 | 43 | 1.9 | 8 | 39.0 | 120 | 40 | slightly loose | yes |
| 23 | 16 | 34 | 3 | 1 | 38.1 | 160 | 40 | physiologic | - |
| 25 | 16 | 49 | 3 | 9 | 39.1 | 140 | 40 | physiologic | - |
| 26 | 15 | 33 | 1.5 | 9 | 38.9 | 108 | 36 | physiologic | - |
| 27 | 15 | 44 | 3 | 1 | 39.2 | 160 | 40 | physiologic | yes |
| 28 | 14 | 44 | 2.5 | 9 | 39.2 | 160 | 40 | physiologic | - |
| 30 | 14 | 45 | 0.5 | 1 | 38.8 | 160 | 32 | slightly solid | - |
| 31 | 14 | 50 | 1.5 | 8 | 38.2 | 160 | 44 | slightly loose | - |
| 32 | 19 | 50 | nA | 8 | 39.1 | 140 | 40 | slightly loose | - |
| 37 | 15 | 38 | 3 | 7 | 39.1 | 120 | 36 | slightly loose | - |
| 40 | 15 | 38 | nA | 8 | 39.4 | 140 | 40 | physiologic | - |

**Continuing Appendix 1:**

| **Findings in Female Calves** | | | | | | | | | |
| --- | --- | --- | --- | --- | --- | --- | --- | --- | --- |
| **Number** | **Age (d)^1^** | **BW^2^** | **Colostrum^3^** | **Housing** | **T**^4^ | **HR**^5^ | **RR**^6^ | **Faeces^7^** | **Umbilical hernia** |
| 45 | 20 | 40 | 2 | 6 | 38.7 | 140 | 44 | physiologic | - |
| 46 | 14 | 43 | 2 | 7 | 38.9 | 160 | 48 | physiologic | - |
| 47 | 18 | 44 | 2 | 4 | 38.1 | 160 | 44 | physiologic | - |
| 48 | 17 | 44 | 3 | 6 | 39.0 | 160 | 36 | physiologic | - |
| 49 | 17 | 43 | 2 | 6 | 39.4 | 160 | 36 | physiologic | - |
| 52 | 21 | 30 | 2.5 | 6 | 38.7 | 160 | 44 | physiologic | - |
| 53 | 20 | 30 | 2 | 6 | 39.3 | 156 | 40 | physiologic | - |
| 54 | 19 | 32 | 2 | 6 | 39.2 | 160 | 44 | physiologic | - |
| 57 | 20 | 30 | 2 | 1 | 38.6 | 148 | 32 | slightly solid | - |
| 61 | 17 | 38 | 3 | 1 | 38.8 | 160 | 40 | physiologic | - |
| 62 | 15 | 36 | 1.5 | 1 | 38.4 | 160 | 40 | physiologic | - |
| 63 | 14 | 48 | nA | 1 | 39.2 | 140 | 32 | physiologic | - |
| 64 | 21 | 50 | 2 | 1 | 38.9 | 120 | 24 | slightly solid | - |
| 66 | 20 | 40 | 2 | 1 | 39.2 | 176 | 32 | physiologic | - |
| 67 | 20 | 45 | 2 | 1 | 38.9 | 160 | 40 | physiologic | - |
| 70 | 15 | 38 | 1.5 | 1 | 38.6 | 104 | 44 | slightly loose | - |
| 72 | 21 | 42 | 2.5 | 6 | 38.6 | 124 | 40 | physiologic | yes |
| 75 | 16 | 43 | 1.5 | 3 | 38.7 | 100 | 32 | physiologic | - |
| 77 | 19 | 42 | 3.5 | 6 | 39.0 | 120 | 40 | physiologic | - |
| 78 | 19 | 34 | 1 | 6 | 39.2 | 120 | 36 | physiologic | - |
| 80 | 16 | 41 | 2 | 8 | 39.5 | 120 | 40 | physiologic | - |
| 81 | 16 | 39 | 2.5 | 3 | 39.2 | 140 | 28 | physiologic | yes |
| 83 | 17 | 44 | 1 | 4 | 38.8 | 140 | 36 | physiologic | - |
| 84 | 14 | 54 | 2 | 8 | 38.8 | 124 | 28 | physiologic | - |

**Continuing Appendix 1:**

| **Findings in Female Calves** | | | | | | | | | |
| --- | --- | --- | --- | --- | --- | --- | --- | --- | --- |
| **Number** | **Age (d)^1^** | **BW^2^** | **Colostrum^3^** | **Housing** | **T**^4^ | **HR**^5^ | **RR**^6^ | **Faeces^7^** | **Umbilical hernia** |
| 85 | 15 | 44 | 4 | 3 | 39.2 | 120 | 28 | physiologic | - |

^1^Age in days. ^2^Body Weight. ^3^Colostrum Intake in liters. ^4^Temperature. ^5^Heart Rate in beats per minute. ^6^Respiratory Rate in breaths per minute. ^7^Consistency of Faeces
