# Supplementary material for: Comparison of plasma substance P concentrations in the blood of healthy male and female German Simmental calves
Source: BMC Vet Res. 2024 May 24;20:226. doi: 10.1186/s12917-024-04010-1 (PMC11127320; doi:10.1186/s12917-024-04010-1)
Supplement: Supplementary file 2 — Supplementary Material 2 [file 12917_2024_4010_MOESM2_ESM.docx]

**Appendix 2:** Laboratory parameters and plasma substance P concentrations (PSPC) in 44 healthy male and 49 healthy female calves of the German Simmental breed. Reference ranges for laboratory parameters as defined by the Clinic for Ruminants with Ambulatory and Herd Health Services are given in brackets. Laboratory findings Laboratory findings were within the reference ranges in 18 (19.4%) of calves. Animals were accordingly divided into two groups, PHYS (no deviations from laboratory parameters) and MDEV (mild deviations from laboratory parameters). All animals were exposed to the same surroundings, feeding, management, and handling.

| **Male calves** | | | | | | | |
| --- | --- | --- | --- | --- | --- | --- | --- |
| **Number** | **Leucocyte count**  (4 – 10 x10³/µl) | **PCV**^1^  (30 – 36%) | **Hemoglobin**  (10 – 13 g/dL) | **Total Protein**  (40 – 80 g/l) | **GSPHX**^2^  (>250 g/Hb) | **PSPC**  (pg/ml) | **Group** |
| 1 | 12.20 | 11.70 | 35.66 | 591 | 57.80 | 451 | MDEV |
| 3 | 5.28 | 10.90 | 34.12 | 436 | 60.80 | 302 | PHYS |
| 4 | 9.75 | 12.20 | 38.55 | 490 | 65.20 | 319 | MDEV |
| 5 | 9.33 | 11.30 | 35.54 | 464 | 56.70 | 422 | PHYS |
| 6 | 7.88 | 10.10 | 31.58 | 571 | 55.80 | 436 | PHYS |
| 7 | 9.20 | 10.80 | 35.54 | 438 | 52.50 | 423 | PHYS |
| 8 | 6.96 | 11.20 | 36.14 | 578 | 55.60 | 418 | MDEV |
| 9 | 5.28 | 10.40 | 33.26 | 525 | 52.60 | 395 | PHYS |
| 11 | 12.60 | 11.90 | 37.75 | 613 | 50.60 | 490 | MDEV |
| 13 | 6.38 | 12.40 | 35.49 | 534 | 50.40 | 367 | PHYS |
| 14 | 7.18 | 11.50 | 36.26 | 411 | 58.80 | 456 | MDEV |
| 15 | 14.29 | 11.20 | 34.02 | 706 | 64.20 | 416 | MDEV |
| 16 | 11.39 | 11.20 | 36.34 | 426 | 52.00 | 558 | MDEV |
| 17 | 8.75 | 11.50 | 37.25 | 588 | 58.40 | 453 | MDEV |
| 18 | 14.20 | 11.60 | 36.26 | 330 | 48.80 | 422 | MDEV |
| 23 | 10.21 | 10.70 | 35.82 | 406 | 47.50 | 501 | MDEV |
| 26 | 7.98 | 12.10 | 38.64 | 491 | 55.50 | 525 | MDEV |
| 31 | 12.56 | 11.00 | 32.31 | 527 | 47.80 | 932 | MDEV |
| 32 | 13.88 | 12.80 | 36.28 | 412 | 54.90 | 743 | MDEV |

**Continuing Appendix 1:**

| **Number** | **Leucocyte count**  (4 – 10 x10³/µl) | **PCV**^1^  (30 – 36%) | **Hemoglobin**  (10 – 13 g/dL) | **Total Protein**  (40 – 80 g/l) | **GSPHX**^2^  (>250 g/Hb) | **PSPC**  (pg/ml) | **Group** |
| --- | --- | --- | --- | --- | --- | --- | --- |
| 33 | 10.99 | 11.20 | 33.44 | 451 | 60.50 | 779 | MDEV |
| 34 | 7.09 | 9.90 | 29.61 | 456 | 52.50 | 676 | MDEV |
| 35 | 8.80 | 12.30 | 36.25 | 401 | 62.60 | 550 | MDEV |
| 36 | 10.28 | 10.50 | 32.33 | 580 | 60.70 | 742 | MDEV |
| 37 | 7.82 | 9.60 | 28.54 | 583 | 63.20 | 652 | MDEV |
| 38 | 14.89 | 12.10 | 34.46 | 377 | 61.00 | 1,129 | MDEV |
| 39 | 14.62 | 10.40 | 32.04 | 678 | 54.10 | 1,057 | MDEV |
| 41 | 10.95 | 11.40 | 35.46 | 705 | 59.80 | 510 | MDEV |
| 45 | 5.51 | 10.90 | 32.23 | 624 | 56.90 | 455 | PHYS |
| 47 | 8.19 | 11.90 | 34.77 | 656 | 49.30 | 974 | PHYS |
| 51 | 7.57 | 11.90 | 36.94 | 572 | 71.40 | 584 | MDEV |
| 54 | 7.68 | 12.00 | 38.98 | 666 | 57.80 | 525 | MDEV |
| 55 | 5.38 | 13.10 | 39.95 | 618 | 56.80 | 549 | MDEV |
| 56 | 12.16 | 11.90 | 37.03 | 616 | 59.30 | 799 | MDEV |
| 57 | 9.18 | 11.80 | 37.57 | 715 | 57.00 | 1,255 | MDEV |
| 58 | 11.70 | 12.20 | 36.73 | 617 | 60.10 | 526 | MDEV |
| 60 | 11.87 | 12.80 | 39.27 | 717 | 59.60 | 651 | MDEV |
| 61 | 10.84 | 12.60 | 38.35 | 749 | 62.00 | 609 | MDEV |
| 63 | 11.02 | 12.5 | 38.55 | 669 | 56.40 | 907 | MDEV |
| 64 | 12.39 | 11.80 | 37.07 | 576 | 58.10 | 867 | MDEV |
| 67 | 9.15 | 12.30 | 39.36 | 823 | 51.20 | 864 | MDEV |
| 70 | 11.57 | 11.30 | 35.55 | 638 | 71.80 | 935 | MDEV |
| 71 | 13.65 | 11.00 | 35.99 | 905 | 54.80 | 739 | MDEV |
| 72 | 9.40 | 10.00 | 32.20 | 715 | 60.90 | 859 | PHYS |
| 73 | 13.03 | 10.00 | 32.32 | 995 | 56.20 | 1,615 | MDEV |

**Continuing Appendix 1:**

| **Female calves** | | | | | | | |
| --- | --- | --- | --- | --- | --- | --- | --- |
| **Number** | **Leucocyte count**  (4 – 10 x10³/µl) | **PCV**^1^  (30 – 36%) | **Hemoglobin**  (10 – 13 g/dL) | **Total Protein**  (40 – 80 g/l) | **GSPHX**^2^  (>250 g/Hb) | **PSPC**  (pg/ml) | **Group** |
| 1 | 9.48 | 12.00 | 36.51 | 367 | 49.50 | 508 | MDEV |
| 4 | 11.91 | 11.00 | 34.04 | 650 | 57.00 | 366 | MDEV |
| 6 | 12.27 | 12.20 | 37.73 | 571 | 53.30 | 246 | MDEV |
| 9 | 12.39 | 12.3 | 37.64 | 401 | 61.80 | 272 | MDEV |
| 10 | 8.95 | 9.4 | 30.42 | 425 | 58.40 | 386 | MDEV |
| 11 | 3.20 | 10.9 | 35.07 | 432 | 63.70 | 236 | MDEV |
| 12 | 11.87 | 12.9 | 38.71 | 372 | 67.10 | 486 | MDEV |
| 13 | 12.27 | 12.9 | 38.21 | 340 | 62.80 | 499 | MDEV |
| 14 | 13.44 | 10.4 | 31.49 | 582 | 58.00 | 475 | MDEV |
| 15 | 9.88 | 11.2 | 34.75 | 618 | 50.60 | 385 | PHYS |
| 16 | 8.48 | 11.7 | 35.36 | 474 | 50.60 | 527 | PHYS |
| 18 | 7.46 | 11.2 | 35.55 | 602 | 66.40 | 271 | PHYS |
| 19 | 12.59 | 9.9 | 33.23 | 615 | 56.90 | 273 | MDEV |
| 21 | 10.45 | 10.7 | 34.87 | 529 | 53.90 | 360 | MDEV |
| 23 | 9.82 | 11.3 | 35.17 | 668 | 58.60 | 472 | PHYS |
| 25 | 9.90 | 12.0 | 36.32 | 370 | 55.20 | 462 | MDEV |
| 26 | 5.42 | 11.8 | 36.52 | 450 | 47.70 | 289 | MDEV |
| 27 | 8.99 | 11.9 | 38.06 | 430 | 52.60 | 475 | MDEV |
| 28 | 11.73 | 10.6 | 33.9 | 482 | 60.10 | 667 | MDEV |
| 30 | 7.33 | 11.8 | 37.30 | 507 | 47.60 | 433 | MDEV |
| 31 | 9.73 | 12.8 | 39.72 | 491 | 59.40 | 839 | MDEV |
| 32 | 11.43 | 11.3 | 35.09 | 297 | 62.20 | 358 | MDEV |
| 37 | 6.14 | 9.5 | 29.78 | 458 | 55.20 | 407 | MDEV |
| 40 | 3.92 | 10.5 | 36.68 | 408 | 53.20 | 470 | MDEV |
| 45 | 12.39 | 10.8 | 31.41 | 642 | 57.40 | 229 | MDEV |
| 46 | 13.45 | 11.7 | 34.07 | 572 | 56.60 | 499 | MDEV |

**Continuing Appendix 1:**

| **Number** | **Leucocyte count**  (4 – 10 x10³/µl) | **PCV**^1^  (30 – 36%) | **Hemoglobin**  (10 – 13 g/dL) | **Total Protein**  (40 – 80 g/l) | **GSPHX**^2^  (>250 g/Hb) | PSPC  (pg/ml) | **Group** |
| --- | --- | --- | --- | --- | --- | --- | --- |
| 47 | 8.83 | 10.1 | 29.13 | 519 | 53.50 | 540 | MDEV |
| 48 | 6.36 | 10.1 | 30.39 | 498 | 54.20 | 489 | PHYS |
| 49 | 12.44 | 12.6 | 34.87 | 602 | 69.40 | 355 | MDEV |
| 52 | 4.20 | 10.5 | 31.59 | 549 | 54.50 | 787 | PHYS |
| 53 | 5.64 | 9.0 | 27.85 | 527 | 54.50 | 516 | MDEV |
| 54 | 5.81 | 11.4 | 31.96 | 396 | 61.30 | 494 | PHYS |
| 57 | 9.27 | 11.9 | 36.44 | 448 | 55.20 | 537 | MDEV |
| 61 | 5.97 | 12.3 | 37.69 | 586 | 65.70 | 237 | MDEV |
| 62 | 8.59 | 9.9 | 30.47 | 643 | 57.10 | 605 | MDEV |
| 63 | 9.15 | 11.1 | 33.58 | 870 | 61.00 | 331 | PHYS |
| 64 | 10.19 | 12.4 | 39.70 | 667 | 54.10 | 640 | MDEV |
| 66 | 9.64 | 11.5 | 34.46 | 473 | 55.80 | 755 | PHYS |
| 67 | 10.33 | 11.3 | 36.13 | 542 | 55.60 | 529 | MDEV |
| 70 | 14.67 | 12.3 | 37.31 | 637 | 52.50 | 409 | MDEV |
| 72 | 10.85 | 11.5 | 35.54 | 828 | 56.8 | 754 | MDEV |
| 75 | 3.34 | 12.2 | 38.31 | 772 | 55.70 | 591 | MDEV |
| 77 | 13.28 | 12.5 | 38.05 | 753 | 56.80 | 704 | MDEV |
| 78 | 8.68 | 12.4 | 37.23 | 658 | 60.00 | 876 | MDEV |
| 80 | 9.43 | 12.6 | 38.88 | 640 | 57.30 | 650 | MDEV |
| 81 | 11.04 | 12.30 | 38.13 | 677 | 56.90 | 946 | MDEV |
| 83 | 9.09 | 11.5 | 38.24 | 701 | 57.30 | 789 | MDEV |
| 84 | 13.65 | 11.0 | 35.99 | 984 | 53.90 | 1,069 | MDEV |
| 85 | 13.03 | 10.0 | 34.70 | 882 | 64.10 | 1,263 | MDEV |

^1^Packed Cell Volume, ^2^Gluthathione Peroxidase
